# Nonlinear analysis of hollow reinforced high strength concrete beams with web openings and different types of stirrups under torsion

**DOI:** 10.1038/s41598-025-32971-2

**Published:** 2026-01-10

**Authors:** Fatma A. Ebrahim, Hamed S. Askar, Mohamed E. El-Zoughiby

**Affiliations:** 1 Structural Engineering Department, Faculty of Engineering, Mansoura University, Mansoura, Egypt; 2 Structural Engineering Department, Mansoura University, Mansoura, 35516 Egypt

**Keywords:** Nonlinear FE analysis, Torsional behavior, Hollow RC beams, High strength concrete, Web openings, GFRP stirrups, Engineering, Civil engineering

## Abstract

In this research, a nonlinear finite element analysis was conducted to investigate the effect of large web openings on the torsional behavior of high-strength hollow reinforced concrete (RC) beams under different transverse reinforcement scenarios. The study presents an extensive numerical program comprising eighty-four specimens modeled with various parameters to develop a wide range of web opening dimensions and transverse reinforcement scenarios. The considered parameters included the web opening size (length and height), transverse reinforcement type (steel, glass fiber reinforced polymer—GFRP—, or no stirrups), transverse reinforcement ratio, longitudinal reinforcement ratio, and concrete strength. All specimens had a constant cross section of 300 × 400 mm, and a total length of 2400 mm. The numerical results revealed that introducing web openings in hollow concrete significantly reduces their torsional capacity by about 48.35%, 46.20%, and 37.58% for beams with concrete strengths of 60 MPa, 80 MPa, and 100 MPa, respectively. Additionally, reducing stirrup spacing and increasing both stirrup diameter and longitudinal reinforcement ratio had a considerable effect in improving torsional capacity. Furthermore, a significant improvement—nearly double—of torsional capacity of hollow beams was observed when using GFRP stirrups as a transverse reinforcement system. Moreover, two numerical equations were adopted to determine the torsional capacity of both the hollow beams considered in the present study and solid beams from other experimental work. The compatibility between the outputs of the proposed equations and the experimental results of others showed reasonable agreement.

## Introduction

Torsion has emerged as a significant issue, acting as a major factor in the brittle failure of structural elements and potentially leading to serious building catastrophes. It can be generated in several cases, such as twisting moments produced by vertical loads on spandrel beams, curved girders and both straight and curved box girders in bridges, as well as RC canopies. Because of their structural efficiency, enhanced stability, serviceability, relatively low construction cost, and appealing aesthetics, hollow beams have gained wide recognition, especially in highway and bridge systems. They are also used to accommodate electrical and mechanical facilities such as water, sewage, air conditioning, electrical power, telephone, and computer networks.

Despite these advantages, hollow RC beams often require transverse openings for maintenance access or to accommodate intersecting duct. Such openings disrupt the normal flow of stresses, producing significant stress concentrations around the opening edges, which in turn lead to premature concrete cracking and substantial reduction in torsional stiffness and capacity. This behavior underscores the necessity for strengthening strategies or alternative reinforcement systems to mitigate the adverse effects associated with web openings.

 Finite element (FE) analysis tools such as ANSYS and ABAQUS offer effective means to examine the torsional behavior of RC beams. A nonlinear FE analysis of RC beams with openings subjected to torsion was conducted by Meleka etal^1^., who reported that increasing the opening length to 50% of the beam depth led to a decrease in ultimate torque by about 15.5%. Similarly, reducing the opening depth by 0.05 of the beam cross section increased the ultimate torque by about 7%. Al-Sherrawi and Shanshal^2^ analyzed RC T-girders with and without openings, and Jabber etal^3^. modeled hollow beams with varying opening sizes under torsional, flexural, and cyclic loads using high-strength concrete (HSC) and ultra-high performance concrete (UHPC). Additional analytical and experimental studies examined beams with large openings strengthened using carbon fiber reinforced polymer (CFRP), glass fiber reinforced polymer (GFRP), or steel plate retrofitting systems^4–9^. Khalil etal^10^. evaluated prestressing techniques for torsional enhancement, while Alamili and Abdulameer^11^ tested hollow I-sections containing square and circular openings constructed from reactive powder concrete (RPC). Further contributions highlighted the influence of repeated loading, high-strength concretes, and innovative reinforcement detailing on torsional behavior^12–16^. Collectively, these studies confirm that web openings substantially reduce torsional strength and stiffness, but they also demonstrate the potential of advanced materials and strengthening methods—such as CFRP, GFRP, RPC, and prestressing—to mitigate these adverse effects.

Unlike previous studies focused mainly on solid beams or specific strengthening methods, the present study uniquely explores the combined influence of web openings, hollow reinforced concrete beams, and their performance under torsion. Moreover, the use of GFRP stirrups in this context remains largely unexplored. Accordingly, the present work addresses this gap by examining the torsional response of these beams and providing original insights not covered in earlier research.

## Research objective

The main objective of this study is to numerically investigate the effect of large rectangular web openings—highlighted by Mansur^17^—on the torsional behavior of high-strength hollow RC beams. The analysis covers various opening lengths and depths, providing the first extensive evaluation of such hollow beams under pure torsion. The study also examines, for the first time, the torsional failure of these hollow beams reinforced internally with GFRP stirrups, rather than with external or near-surface GFRP mounted rods, wraps or fibers mixed into the concrete, as used in previous studies. Based on the numerical results, crack patterns and torsional capacities will be determined, and a predictive equation proposed to reliably estimate experimental behavior.

## Numerical study

### ANSYS model verification

To identify the optimal mesh density, three mesh sizes—90 mm, 50 mm, and 30 mm—were evaluated for beam BH_o_−60Mpa tested by Ahmed^18^ and beam CBO tested by Eltaly etal^13^.. The torsional capacities computed for each mesh configuration were compared with the corresponding experimental results. Based on this comparison, a mesh size of 50 mm was determined to provide the most accurate and reliable predictions, as summarized in Table 1.

**Fig. 1:**
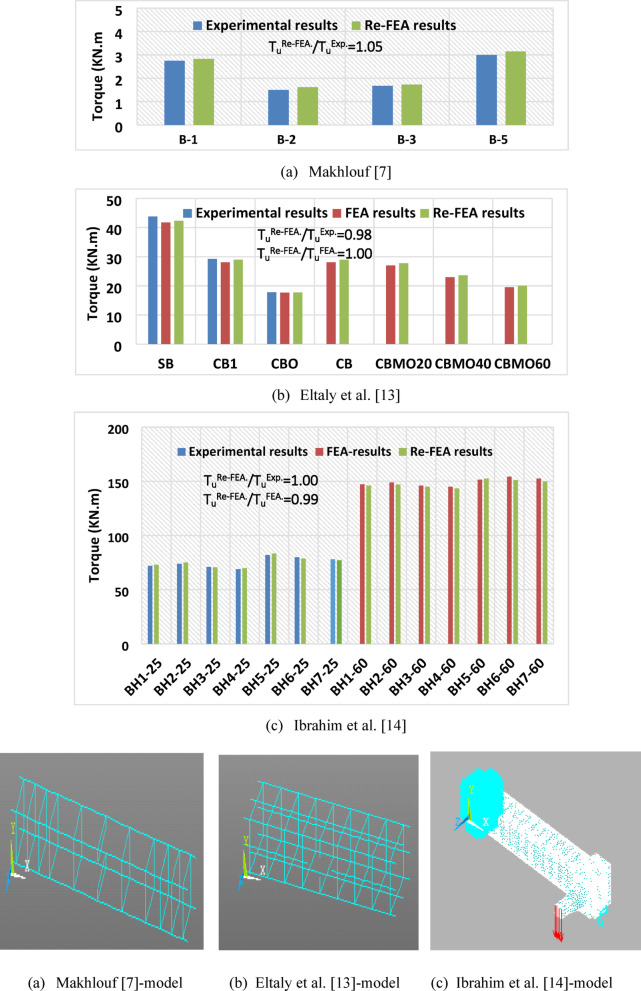
Finite element validation for previous studies using ANSYS software.

The validity of the FE results obtained in the current study was confirmed by re-analyzing 25 previously tested specimens that had been experimentally and numerically investigated in the literature^7,13^, and14] using ANSYS software. Upon re-modeling these specimens, a strong agreement was observed, as shown in Fig. 1. Moreover, one of the reference specimens adopted in the current study (BHM_o_−60 MPa) showed a high level of agreement with its corresponding specimen (BH_o_−60Mpa) reported in Ahmed’s PhD thesis^18^, exhibiting nearly identical properties and dimensions, with an agreement level of 102%.

**Table 1:** Mesh convergence check.

Study	Beam	Torsional capacity, kN.m			
		FEA			
		Mesh sizes, mm			
		30	50	90	Exp.
^13^	CBO	17.52	17.73	18.3	17.8
^18^	BH_o_	137.02	137.2	138.7	137.8

### Parametric study

**Fig. 2:**
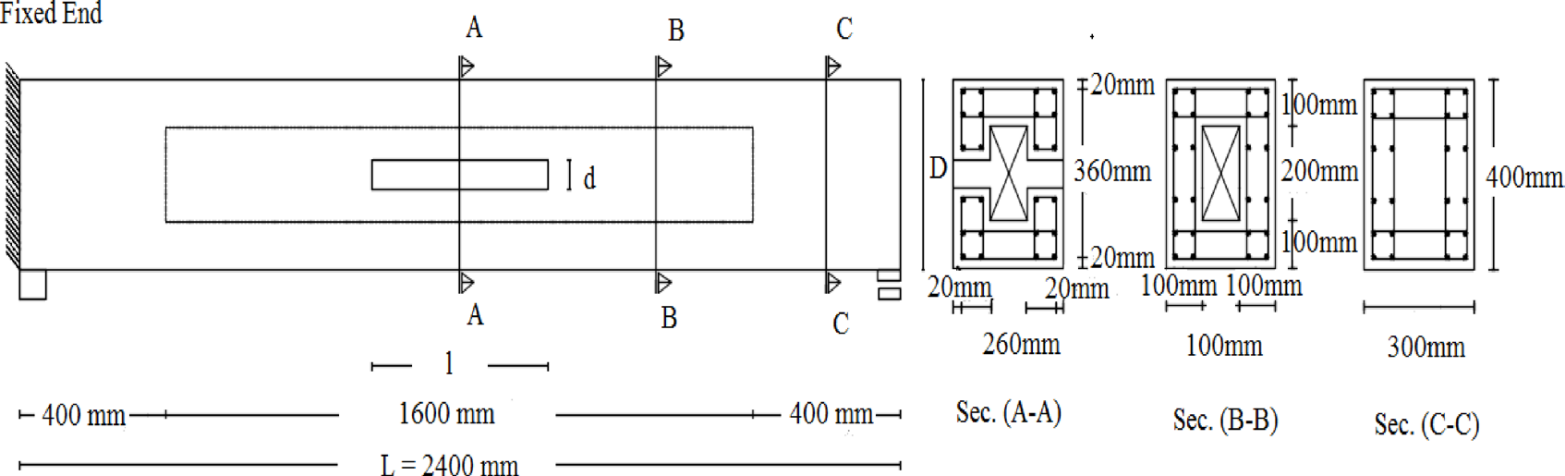
Geometry of numerical investigated specimens.

The numerically simulated beams had the same geometrical and material properties: a 300 × 400 mm rectangular cross-section, a web thickness of 100 mm for the hollow beams, a total length of 2400 mm, and a tested span of 1600 mm, as shown in Fig. 2. The beams were divided into nine groups, one control specimen reinforced with mild steel stirrups and without web openings, served as the reference for groups 1, 2, and 3.

Groups 1 through 3 had the same stirrup spacing S of 160 mm, stirrup diameter D of 6 mm, and longitudinal rebar diameter R of 10 mm, but varied in opening size. The ratios of opening depth to the beam depth and opening length to beam length are denoted as $\:d/D$ and$\:l/L$, respectively. Groups 2-S_2_ and 2-S_3_ refer to the same specimens in Group 2 but with reduced stirrup spacing, (S_2_ = 100 mm and S_3_ = 80 mm). Group 2-D_2_ refers to the same specimens in Group 2 but with an increased stirrup diameter, (D_2_ = 8 mm). Group 2-R_2_ maintains the same specimens but increases the longitudinal rebar diameter, (R_2_ = 12 mm). Group 2-N is identical to Group 2 except that stirrups were removed. Group 2-GFRP replaces the steel stirrups of Group 2 with GFRP stirrups. Analyses of these groups were performed with three concrete grades: 60, 80, and 100 MPa. The detailed properties of the modeled specimens are summarized in Table 2, with a note indicating how the required areas were calculated to determine the transversal and longitudinal reinforcement ratios (*ρ*_st_ and *ρ*_sl_), which were defined following Lopes et al.^19^.

**Table 2:** Specifics of the simulated beams and numerical results.

Group no.	Specimen	Web opening		Transverse rebar			Longitudinal rebar		$\:{T}_{u}$ kN-m		
		$\:\frac{d}{D}$	$\:\frac{l}{L}$	Spacing, mm	Dia., mm	Ratio, $\:{\rho\:}_{st}$%	Rebar	Ratio, $\:{\rho\:}_{sl}$%	$\:{f}_{cu}$, MPa		
									60	80	100
Ref.	BHM_o_	–	–	160	6	0.183	10	1.57	136.09	165.75	186.88
1	BHM1	0.15	0.3	160	6	0.543	10	1.85	107.25	123.5	139.8
	BHM2	0.15	0.525	160	6	0.551	10	1.85	84.5	102.5	1130
	BHM3	0.15	0.75	160	6	0.575	10	1.85	73.45	92.68	118.56
2	BHM4	0.2	0.3	160	6	0.559	10	1.96	91.81	121.06	135
	BHM5	0.2	0.525	160	6	0.567	10	1.96	80	92.63	126.25
	BHM6	0.2	0.75	160	6	0.589	10	1.96	72.46	89.38	115.38
3	BHM7	0.25	0.3	160	6	0.579	10	2.09	82.97	100.75	130
	BHM8	0.25	0.525	160	6	0.585	10	2.09	74.75	89.7	121
	BHM9	0.25	0.75	160	6	0.605	10	2.09	59.07	81.25	107.25
2-S_2_	BHM10	0.2	0.3	100	6	0.886	10	1.96	121.88	135	149.5
	BHM11	0.2	0.525	100	6	0.895	10	1.96	113.75	121.88	140.84
	BHM12	0.2	0.75	100	6	0.907	10	1.96	87.88	107.25	121.88
2-S_3_	BHM13	0.2	0.3	80	6	1.102	10	1.96	125.67	135.78	150.5
	BHM14	0.2	0.525	80	6	1.112	10	1.96	119	126.75	144.86
	BHM15	0.2	0.75	80	6	1.134	10	1.96	96	110.5	126.75
2-D_2_	BHM16	0.2	0.3	160	8	0.995	10	1.96	106.96	133.25	156
	BHM17	0.2	0.525	160	8	1.000	10	1.96	85.31	111.46	131.46
	BHM18	0.2	0.75	160	8	1.050	10	1.96	73.13	106.9	125
2-R_2_	BHM19	0.2	0.3	160	6	0.559	12	2.83	95.75	121.75	148.85
	BHM20	0.2	0.525	160	6	0.567	12	2.83	90.09	109.6	127.29
	BHM21	0.2	0.75	160	6	0.589	12	2.83	83.5	96.08	121.88
2-N	BHN1	0.2	0.3	–	–	–	10	1.96	88.8	110	126.75
	BHN2	0.2	0.525	–	–	–	10	1.96	76.38	83.5	117.23
	BHN3	0.2	0.75	–	–	–	10	1.96	68.58	81.61	93.76
2-GFRP	BHG1	0.2	0.3	160	6	0.559	10	1.96	107.2599.13	133.25	156
	BHG2	0.2	0.525	160	6	0.567	10	1.96	82.55	117.5	139.75
	BHG3	0.2	0.75	160	6	0.589	10	1.96		102.25	126.94

BHM refers to Beam-Hollow-Mild steel stirrup; BHN refers to Beam-Hollow-No stirrups; BHG refers to Beam-Hollow-GFRP stirrup; A_st_ = cross sectional area of all stirrups (in web opening region and out-side it) through the tested length of the beam.

A_c_ = cross sectional area of the tested beam with considering the region of opening (region of opening or not).

A_s_ = cross sectional area of the longitudinal rebar through the tested length of the beam.

## Non-linear FE analysis

### Assumptions and implications

The FE analysis assumed concrete to be homogeneous and isotropic, with perfect bonding between concrete and the reinforcement. Time-dependent effects such as creep and shrinkage were neglected. The Willam–Warnke^20^ failure model was adopted to capture nonlinear concrete behavior in compression and cracking in tension. Reinforcement was modeled with idealized elastic–plastic properties, while GFRP stirrups were considered linearly elastic.

These assumptions were introduced to simplify the analysis and focus on the main effects of large web opening and internal GFRP stirrups, thereby improving numerical stability. However, they may lead to an overestimation of torsional capacity compared with real structural behavior. Future studies could consider more realistic material behavior.

### Modeling

Concrete—with and without reinforcement—was modeled using the SOLID65 element with 8 nodes, each having three degrees of freedom. Steel reinforcement was modeled using the LINK180 three-dimensional uniaxial tension–compression element in ANSYS V-15^21^.

### Materials

#### Concrete

The SOLID65 element was defined by linear isotropic and multi-linear isotropic material properties in ANSYS V-15^21^. ACI 318 − 19^22^ was used to determine the elasticity modulus ***E***_***c***_ for the concrete. Poisson’s ratio υ_c_ was assumed to be 0.2. Desayi and Krishnan^23^ equations, which define the multi-linear isotropic stress–strain relation of concrete, were used to obtain the compressive uniaxial stress–strain values of concrete model:

1 $$\:f\:=\:{E}_{c}\epsilon\:/(1+{(\epsilon\:/{\epsilon\:}_{0})}^{2})\:\text{M}\text{P}\text{a}$$

2 $$\:{{\upepsilon\:}}_{0}=2{f}_{\text{c}}^{{\prime\:}}/{E}_{\text{c}}$$

*f* is the stress (in MPa) at any strain ɛ, ɛ_o_ is the strain corresponding to the peak stress$\:{f}_{\text{c}}^{{\prime\:}}$, and $\:{f}_{\text{c}}^{{\prime\:}}$ is assumed equal to 0.8*f*_*cu*_.

3 $$\:Ec\:=\:3320\sqrt{fc}^{\prime}+6900\:\left(\text{M}\text{P}\text{a}\right),\left(\text{A}\text{C}\text{I}\:318-19,\left[22\right]\right)\:\text{f}\text{o}\text{r}\:\text{H}\text{S}\text{C}$$

The uniaxial tensile cracking stress, *f*_*r*_, is

4 $$\:fr\:=\:0.62\sqrt{fc}^{\prime}\:\left(\text{M}\text{P}\text{a}\right),\:\left(\text{A}\text{C}\text{I}\:318-19,\:\left[22\right]\right)\:$$

The use of the Willam and Warnke^20^ material model in ANSYS requires various constants to be defined, as shown in Table 3.

**Table 3:** Concrete constants for Willam and Wranke material model.

	Concrete constant	C(60)	C(80)	C(100)
1	Shear transfer coefficients for an open crack (*β*_*t*_)	0.30		
2	Shear transfer coefficients for a closed crack (*β*_c_)	0.80		
3	Uniaxial tensile cracking stress (*f*_r_)	4.3 MPa	4.96 MPa	5.55 MPa
4	Uniaxial crushing stress ($\:{f}_{C}^{{\prime\:}}$)	48 MPa	64 MPa	80 MPa

#### Longitudinal and transverse rebar

LINK-180 element requires linear and bilinear- isotropic properties, ANSYS V-15^21^. The elasticity modulus $\:{E}_{s}$ was taken 2×$\:{10}^{5}\:$MPa, the tangent modulus $\:{E}_{t}$ = 0.1$\:{E}_{s}$, Cervenka etal^24^., and Poisson’s ratio υ_s_ was assumed to equal 0.30. Two yield strengths$\:\:\left(\:{f}_{y}\right)$ were considered, $\:{f}_{y}$= 240 MPa for mild steel stirrups and 400 MPa for longitudinal rebar.

#### GFRP stirrups

LINK180 element was used to model the GFRP stirrups, but with only linear properties, following Ahmad etal^25^.. The elasticity modulus was taken 40000 MPa, and Poisson’s ratio was assumed to be 0.20.

### Meshing and boundary conditions

**Fig. 3:**
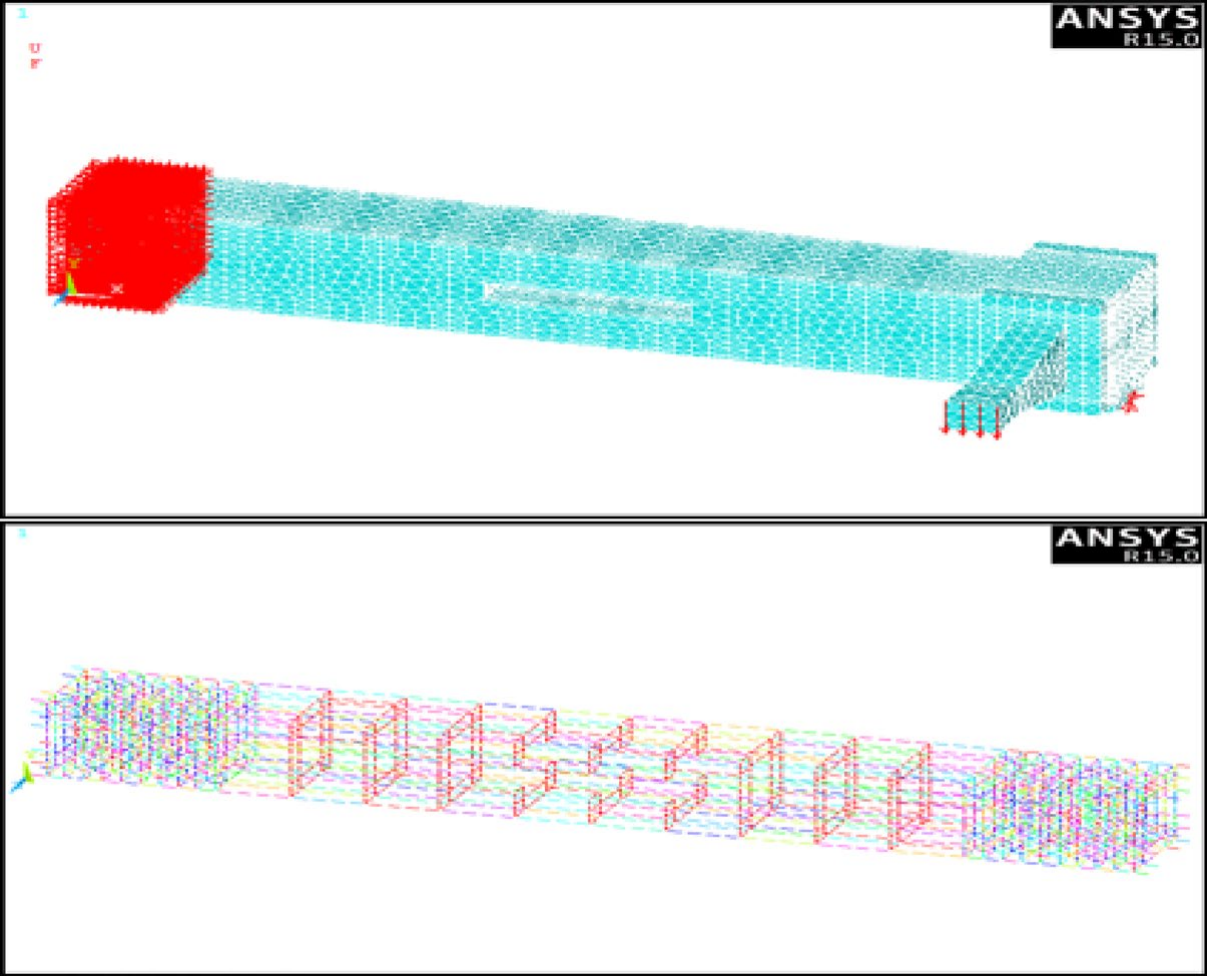
Modeling, boundary conditions and meshing by ANSYS V15.0.

The concrete volumes were meshed using the SOLID-65 element, while the longitudinal reinforcement and stirrups (including both mild steel and GFRP stirrups) were meshed using LINK180 elements. The modeling and meshing process is shown in Fig. 3.

The boundary conditions of the numerically simulated specimens were defined in accordance with Ibrahim etal^14^.. A full restraint was applied at one end of each beam by restraining all translational and rotational degrees of freedom. At the opposite loaded end, the beam was restrained only in the vertical plane, allowing free rotation and axial deformation.

### Non-linear FE results

#### Crack pattern and stress flow

**Fig. 4:**
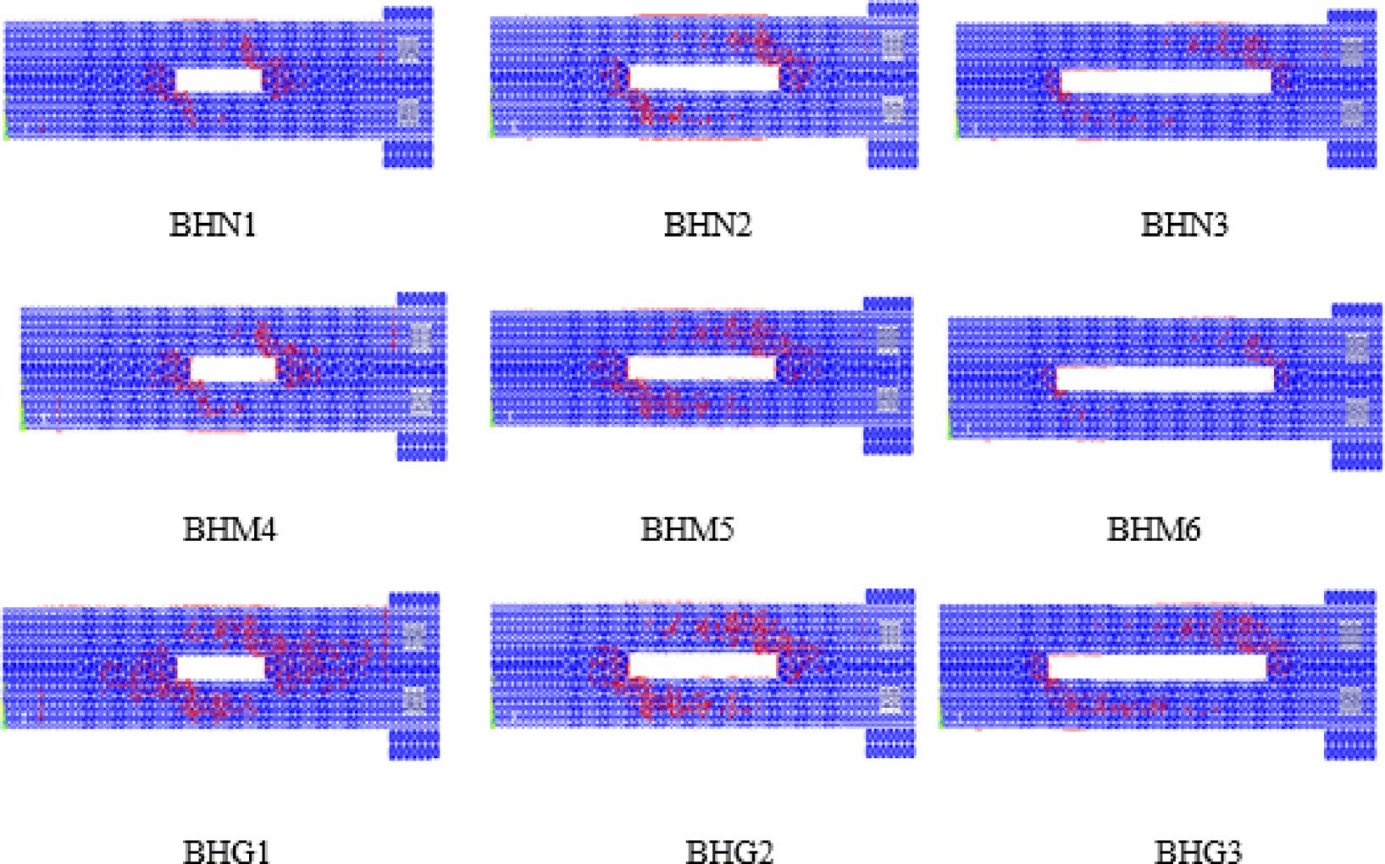
Samples of Initial crack pattern for the simulated beams.

As shown in Fig. 4, cracking initiated at the opening corners due to local stress concentration and then propagated diagonally, forming a spiral pattern. Increasing the opening length intensified crack localization to the corners and further weakened the torsional shear path.

**Fig. 5:**
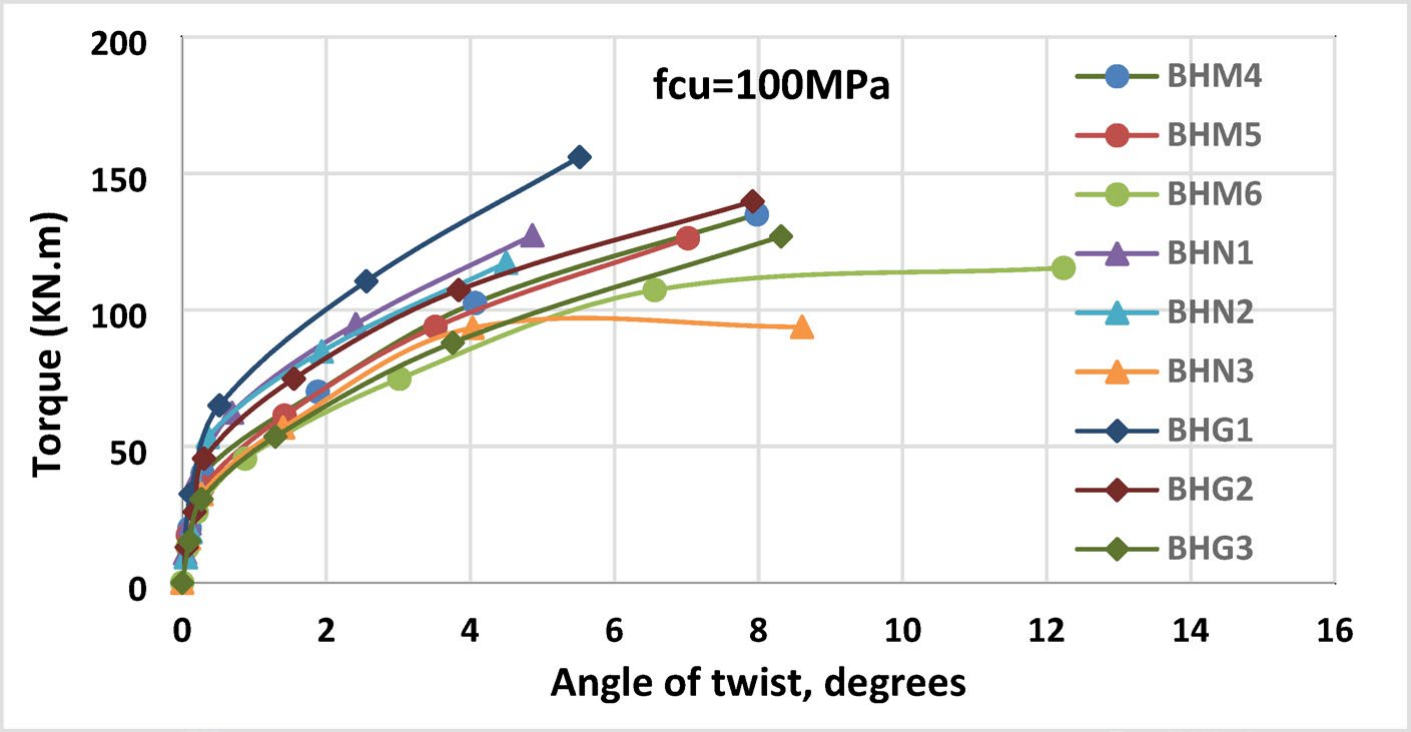
Torque-twist angle correlation of beams of concrete strength of 100 MPa with different systems of stirrups.

Following initial cracking, the reduced slope of torque–twist curves reflects a loss of stiffness caused by larger openings$\:(l/L$ > 0.525), explaining the vulnerability of beam sections around openings under torsional loading, as shown in Fig. 5.

GFRP stirrups were more effective in delaying crack formation and improving post-cracking behavior because their high tensile strength provides better confinement and resistance to diagonal shear stresses.

**Fig. 6:**
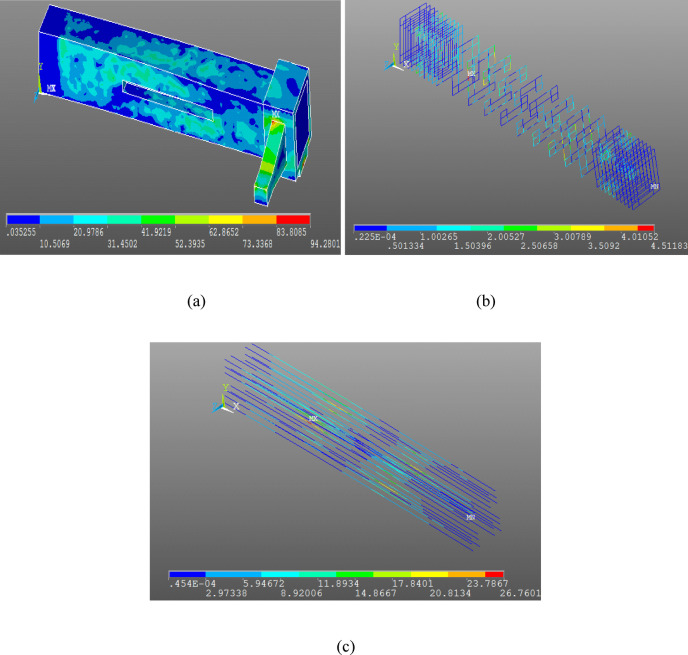
Typical FEA torsional stresses results. (**a**) Concrete outer surface and around opening. (**b**) In stirrups. (**c**) In longitudinal reinforcing steel bars.

As shown in Fig. 6, high stress concentrations occurred along the opening edges and beam corners, defining the main torsional shear flow path. Peak stresses in the stirrups near their junctions with the longitudinal bars emphasized their role in restraining diagonal cracking, while rising stresses in the longitudinal reinforcement confirmed their role in redistributing torsional forces. This stress distribution explains the initiation and propagation of diagonal cracks and the resulting reduction in torsional capacity.

#### Effect of opening size on torsional strength

##### Increasing opening depth (constant opening length)

**Fig. 7:**
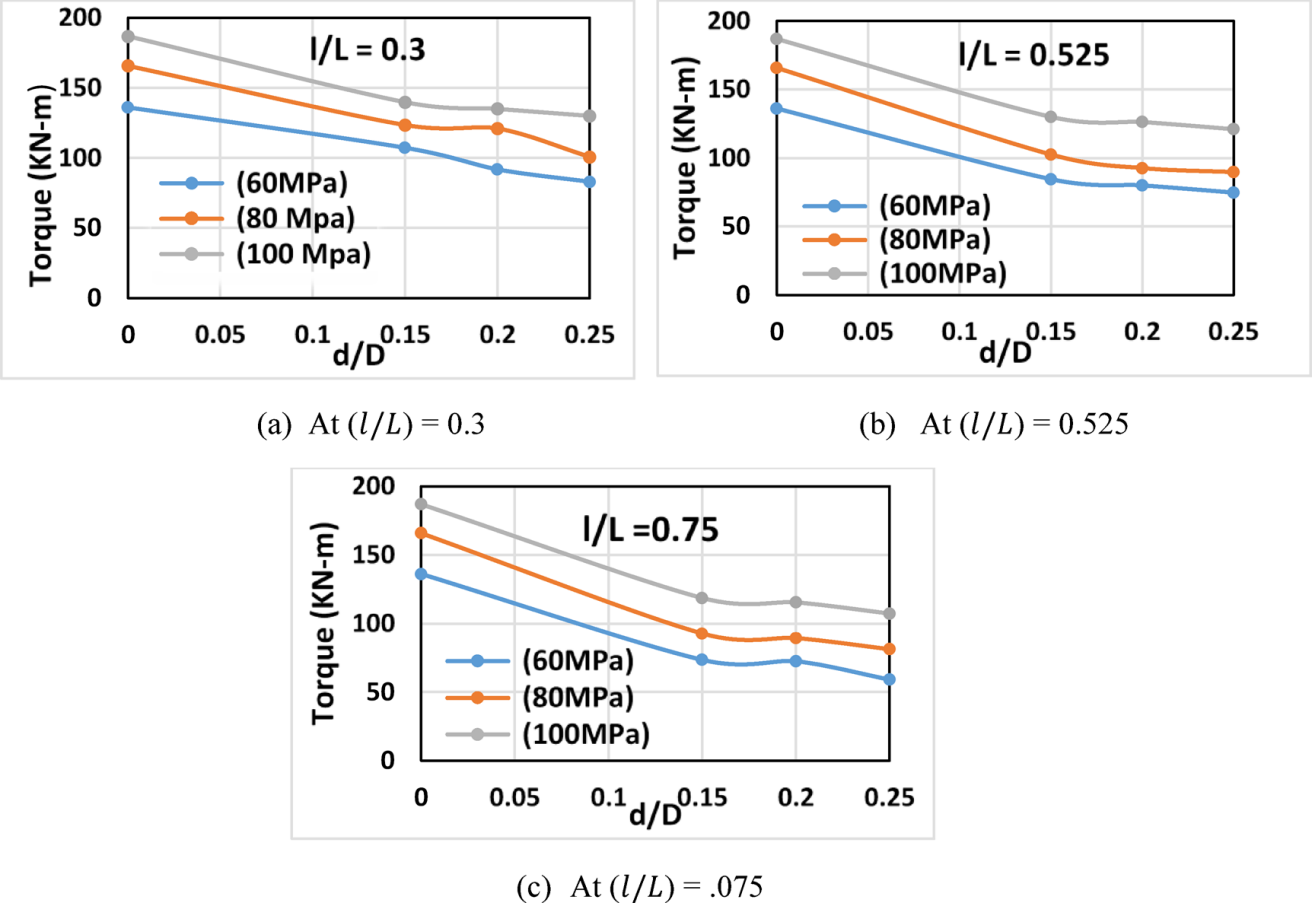
Torque vs. ratio of the opening depth to beam depth (*d/D*) for beams of different concrete strengths.

Increasing the opening depth ratio ($\:d/D)$ from 0 to 0.25 reduced the torsional capacity by approximately 30.4–56.6%, depending on concrete strength and opening length, as shown in Fig. 7. This reduction was more pronounced for longer openings ($\:l/L$ > 0.525), highlighting the significant weakening caused by extended openings which disrupt the torsional stress path more severely. High-strength concrete (100 MPa) partially offset this reduction, improving torsional capacity by about 10%.

##### Increasing opening length (constant opening depth)

**Fig. 8:**
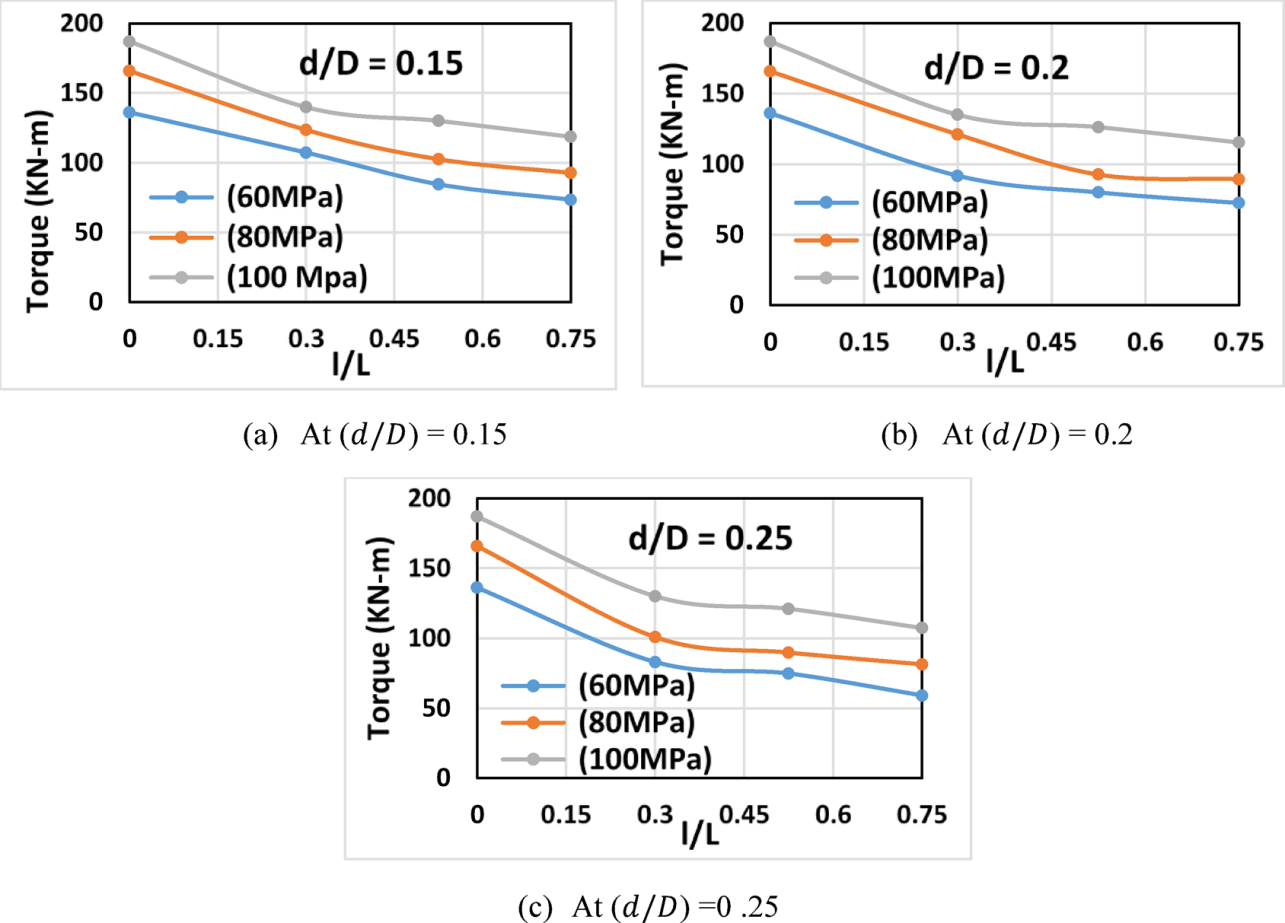
Torque vs. ratio of the opening length to beam length (*l/L*) for beams of different concrete strengths.

Increasing the opening length ratio ($\:l/L$) from 0 to 0.75 decreased torsional capacity by about 36.5–56.6%, depending on concrete strength and opening depth, as shown in Fig. 8. The greatest reduction occurred for deeper openings ($\:d/D$ > 0.2), which enlarge the region of concrete affected by shear and weaken the beam. High-strength concrete (100 MPa) partially compensated for this weakening effect, increasing torsional capacity by about 7%.

#### Effect of transverse steel ratio ($\:{\rho\:}_{st}$)

##### Reducing stirrup spacing

**Fig. 9:**
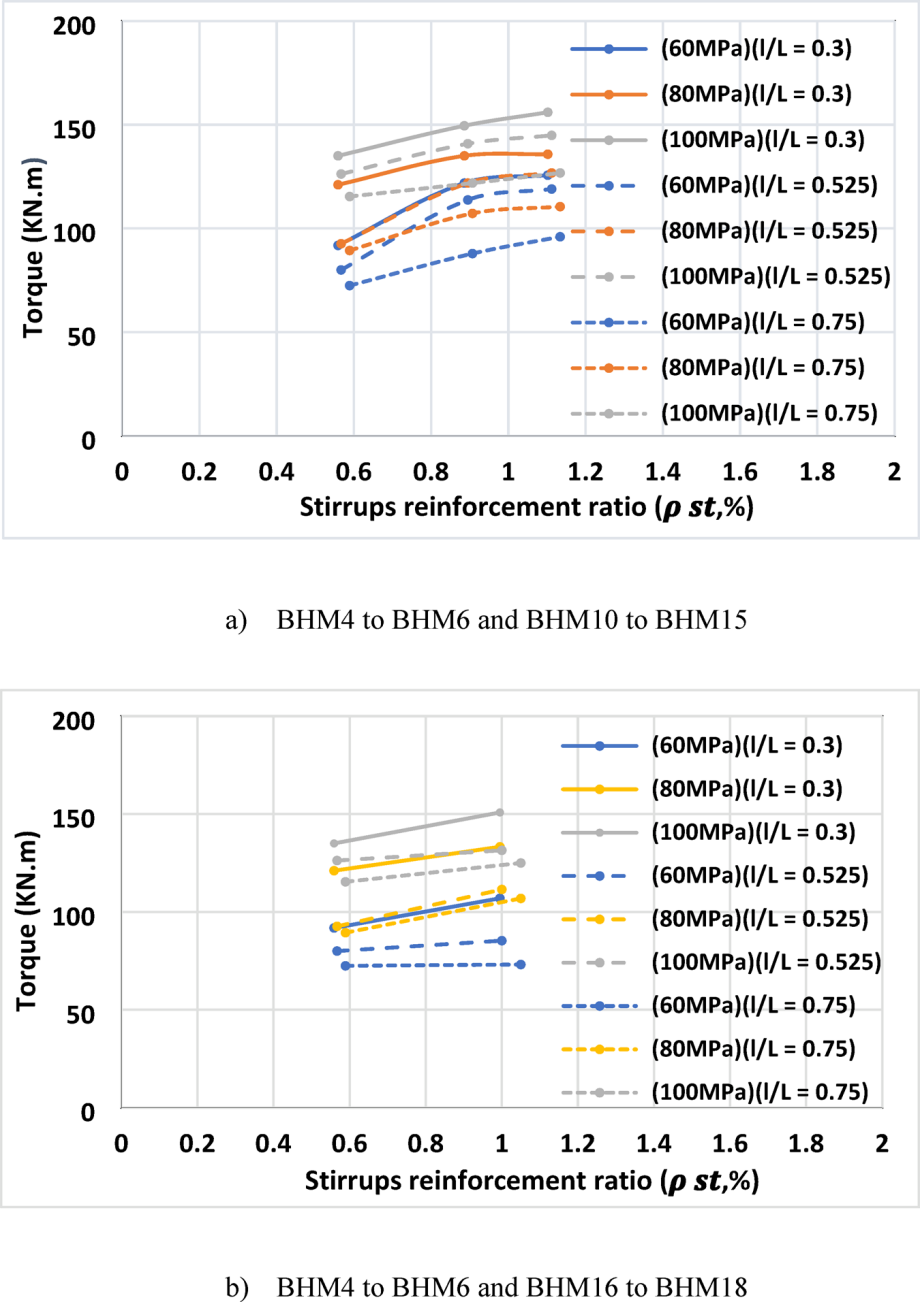
Torque vs. mild transversal stirrups steel ratio for beams of changed concrete strengths.

Reducing stirrup spacing from 160 mm to 80 mm (increasing the stirrup ratio from 0.559% to 1.102%), improved torsional capacity by approximatel**y** 8.97–32.77%, being more effective for short openings and low-strength concrete, as shown in Fig. 9a. its effectiveness decreases for high-strength concrete, which can carry more torsional stresses, making the stirrups’ contribution less significant, and for long openings ($\:l/L$ >0.525) due to early local failures near the openings.

##### Increasing stirrup diameter

Increasing stirrup diameter from 6 mm to 8 mm (increasing the stirrup ratio from 0.317% to 0.563%) raised torsional capacity by approximately 1–16.9%, mostly for smaller openings and lower-strength concrete, as shown in Fig. 9b. For large openings, the effectiveness was limited due to the reduced concrete area around the stirrups, which restricted their anchorage. Moreover, in higher strength concrete, the concrete itself contributed more significantly to torsional resistance, reducing the relative impact of stirrup reinforcement.

#### Effect of longitudinal rebar ratio ($\:{\rho\:}_{sl}$)

**Fig. 10:**
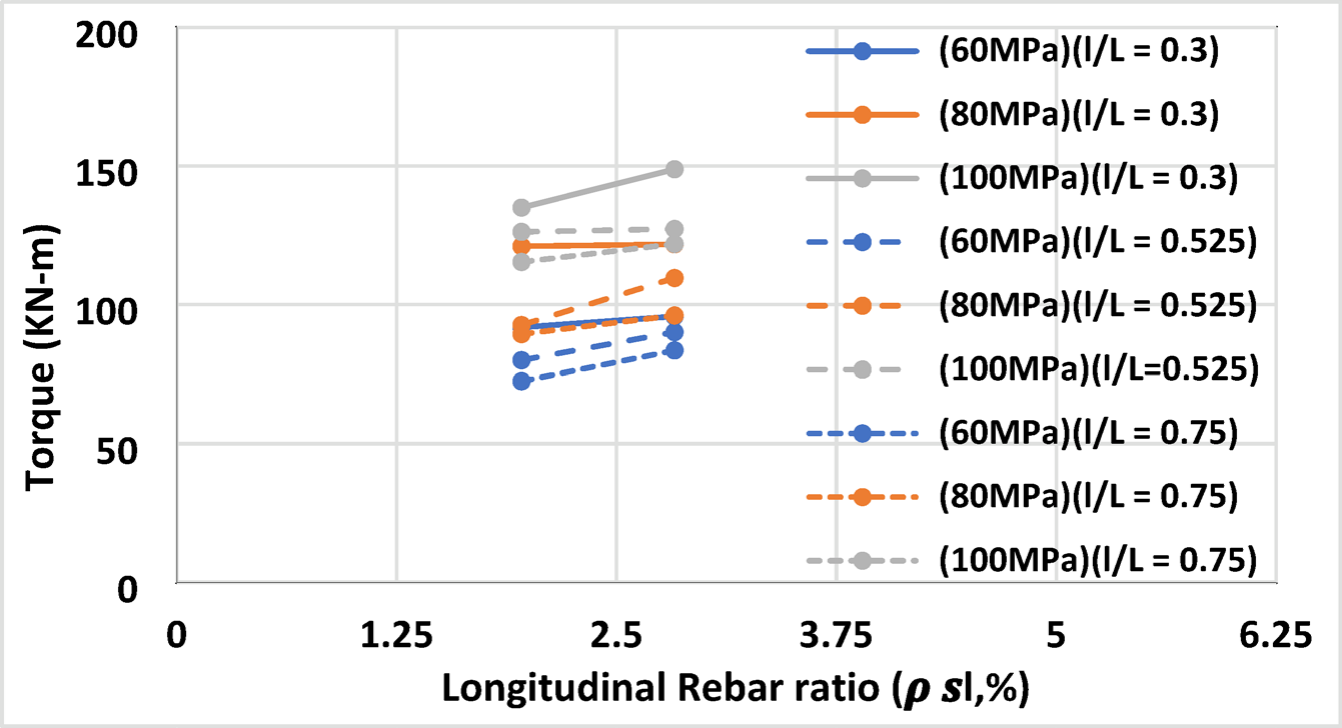
Torque vs. longitudinal rebar ratios of beams (BHM4 to BHM6 and BHM19 to BHM21) of different concrete strengths.

As shown in Fig. 10, increasing the longitudinal reinforcement ratio from 1.96% to 2.83% enhanced torsional capacity by approximately 1–13.22%. This improvement was more pronounced in beams with low-strength concrete and long openings, where additional longitudinal bars played a larger role in restoring torsional strength. In high-strength concrete, the relative impact of extra longitudinal bars was smaller, as the concrete itself provides substantial torsional resistance.

#### Effect of transverse steel type

**Fig. 11:**
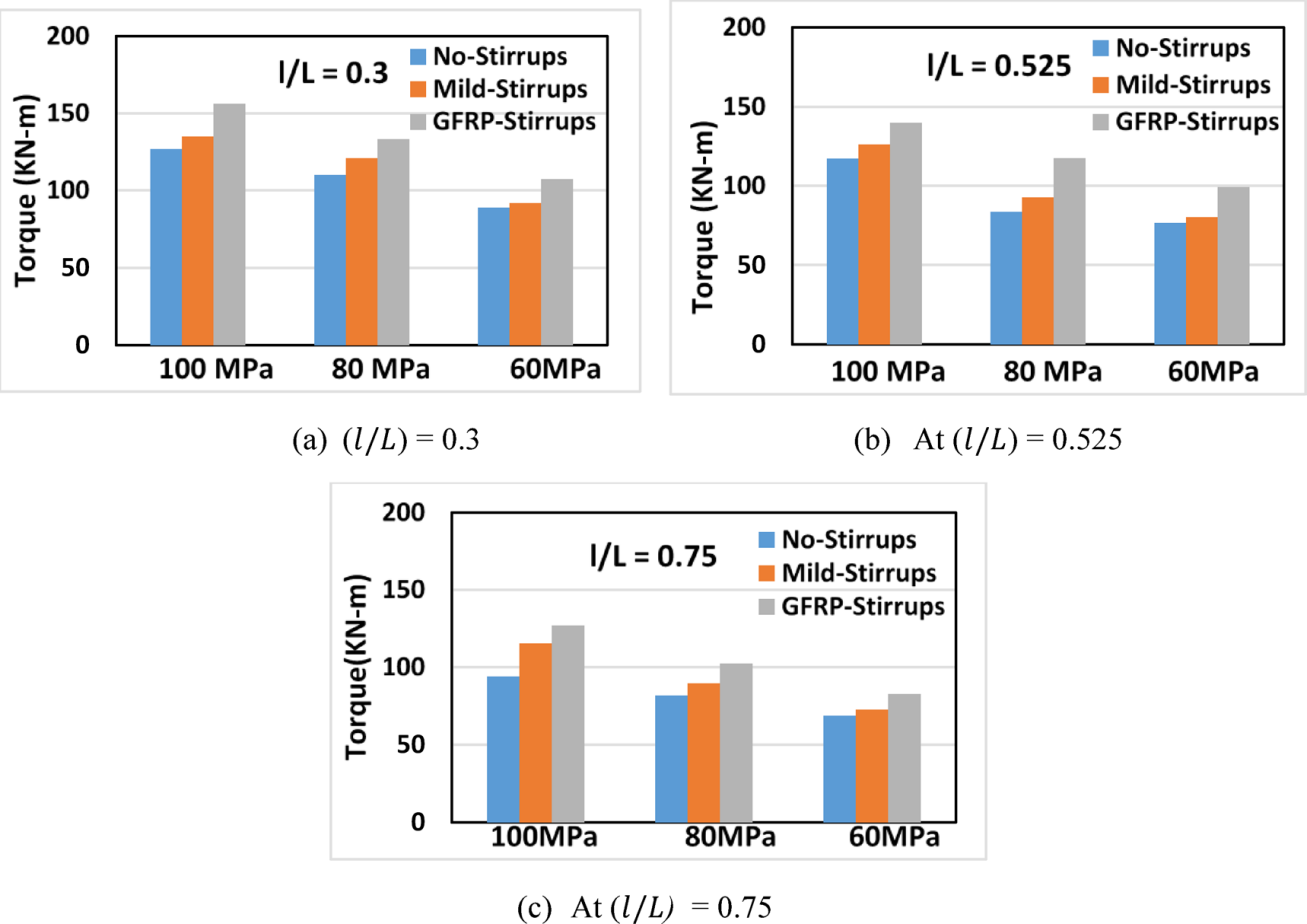
Effect of transverse steel type on torque for beams (BHM4 to BHM6, BHN1 to BHN3, and BHG1 to BHG3) of different concrete strengths.

Figure 11 illustrates the effect of transverse reinforcement on torsional capacity at a constant depth ratio $\:(d/D\:=\:$0.2). Using mild-steel stirrups increased the capacity by approximately 3.3–9.9%, while replacing them with GFRP stirrups further improved capacity by 9.1–21.2%, depending on concrete strength and opening length. GFRP stirrups are more effective than mild steel due to their higher tensile strength, particularly in beams with lower-strength concrete. However, the improvement decreases for long openings ($\:l/L$ > 0.525) because of early local failures limiting stirrup contribution. From a practical perspective, the use of GFRP stirrups involves higher cost, limited availability, specialized handling, and lack of explicit code provision. Tables 4 and 5 summarize previous cost–benefit analysis and durability studies for GFRP applications^26–32^.

**Table 4:** Summary of cost–benefit findings for GFRP.

Study	Initial cost vs. steel	Life-cycle cost savings	Key note
Al Omar and Abdelhadi^26^	lightly higher (not quantified)	Up to ≈ 85% lower total emissions and notable economic savings over full service life	Focus on environmental + economic sustainability
Ascione et al^27^.	≈ 15% higher	26% lower global life-cycle cost due to minimal maintenance	Demonstrates long-term economic viability.
Younis et al^28^.	Similar to steel	≈ 50% lower life-cycle cost over 100 years	Excellent for marine exposure with reduced maintenance.

**Table 5:** Summary of durability studies for GFRP.

Study	Exposure condition	Duration	Tensile strength retention	Key note
Jin et al^29^.	Alkaline solution + sustained load	180 days (accelerated)	~ 56% (worst case)	Loss increases with sustained stress and alkalinity.
Tu et al^30^.	Simulated seawater & concrete pore solution	180 days (lab)	70–90%	Good retention under moderate marine conditions.
Feng et al^31^.	Alkaline + elevated temperature	180–270 days (accelerated)	45–60%	Larger diameter and higher T accelerate degradation.
Myers et al^32^.	In-service bridge cores	17 years (field)	~ 98% (≈ 2% loss)	Excellent long-term performance in real structure.

## Proposed numerical equations

### Derived equation for Hollow RC beams

The proposed equation was developed by incorporating all studied variables into Excel’s Scientific Data Analysis tool to obtain the best-fit linear relationship. The coefficients were then slightly adjusted to better match the torsional moment values ($\:{\varvec{T}}_{\varvec{u}}$ in N·mm), resulting in the final form of the equation:

5 $$\:{T}_{u}={10}^{6}\left[30\left(X\right)+{10}^{-10}\left(Y\right)-305\left(Z\right)\right]$$

6 $$\:X\:=\left(1+1.4\:{\rho\:}_{st}\:\right)$$

7 $$\:{Y\:=t}^{3}\left({236\rho\:}_{sl}.{f}_{y}+12700{f}_{c}-{E}_{st}\right)$$

8 $$\:Z\:=\:\left(\frac{d}{D}x\frac{l}{L}\right)$$

**Fig. 12:**
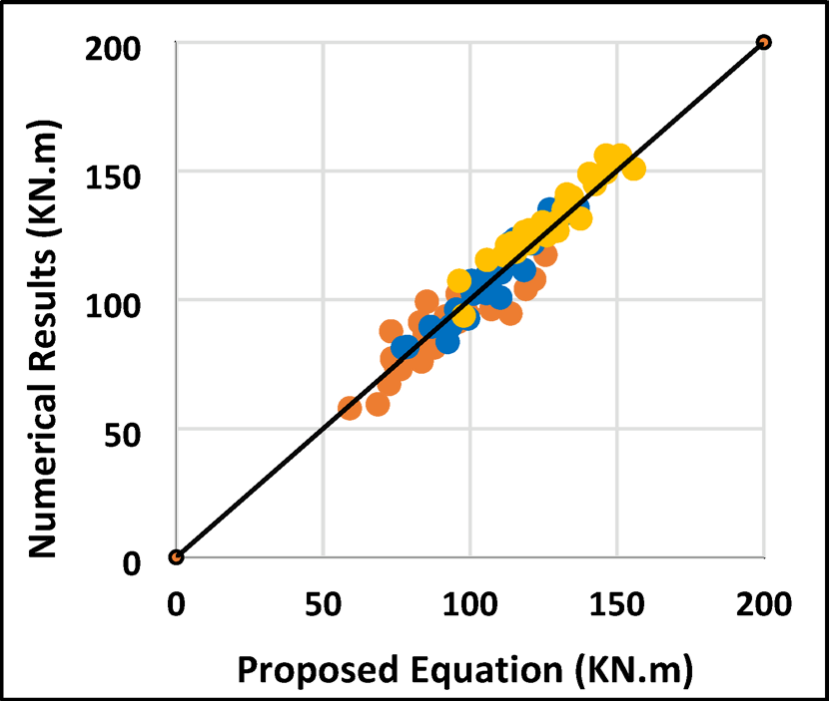
Comparison of the proposed equation results with the numerical results.

In the equation, ***t*** represents the equivalent thickness of the shear flow zone, $\:{\varvec{E}}_{\varvec{s}\varvec{t}}$ is the young’s modulus of the used stirrups; all lengths in mm and all stresses are in MPa. The equation was applied to the simulated specimens and the results, along with standard deviaton (σ) and coefficient of variation (v), are presented in Table 6. A comparison between the equation out-put and the numerical outcomes is shown in Fig. 12, demonstrating good agreement between the model and the simulations.

**Table 6:** Association between the numerical outcomes and the equation out-put for the simulated beams.

Group no.	$\:\:{{T}_{u}}^{FE},\:\text{k}\text{N}.\text{m}$			$\:{{T}_{u}}^{Eqn.},\:\text{k}\text{N}.\text{m}$			$\:\frac{{{T}_{u}}^{Eqn.}}{{{T}_{u}}^{FE}}$		
	60	$\:{f}_{cu}$, MPa 80	100	60	$\:{f}_{cu}$, MPa 80	100	60	$\:{f}_{cu}$, MPa 80	100
1	107.25	123.5	139.8	98.50	119.30	140.101	0.92	0.97	1.00
	84.5	102.5	130	88.54	109.34	130.14325	1.05	1.07	1.00
	73.45	92.68	118.56	79.26	100.06	120.8575	1.08	1.08	1.02
2	91.81	121.06	135	95.61	116.41	137.21	1.04	0.96	1.02
	80	92.63	126.25	82.22	103.02	123.821	1.03	1.11	0.98
	72.46	89.38	115.38	69.42	90.22	111.02	0.96	1.01	0.96
3	82.97	100.75	130	93.07	113.87	134.671	1.12	1.13	1.04
	74.75	89.7	121	76.17	96.97	117.76675	1.02	1.08	0.97
	59.07	81.25	107.25	59.85	80.65	101.4505	1.01	0.99	0.95
2-S_2_	121.88	135	149.5	109.34	130.14	150.944	0.90	0.96	1.01
	113.75	121.88	140.84	96.00	116.80	137.597	0.84	0.96	0.98
	87.88	107.25	121.88	82.78	103.58	124.376	0.94	0.97	1.02
2-S_3_	125.67	135.78	150.82	118.42	139.22	160.016	0.94	1.03	1.06
	119	126.75	144.86	105.11	125.91	146.711	0.88	0.99	1.01
	96	110.5	126.75	92.31	113.11	133.91	0.96	1.02	1.06
2-D_2_	106.96	133.25	156	113.92	134.72	155.522	1.07	1.01	1.00
	85.31	111.46	131.46	100.41	121.21	142.007	1.18	1.09	1.08
	73.13	106.9	125	88.78	109.58	130.382	1.21	1.03	1.04
2-R_2_	95.75	121.75	148.85	103.61	124.41	145.214	1.08	1.02	0.98
	90.09	109.6	127.29	90.23	111.03	131.825	1.00	1.01	1.04
	83.5	96.08	121.88	77.42	98.22	119.024	0.93	1.02	0.98
2-GFRP	107.25	133.25	156	111.61	132.41	153.21	1.04	0.99	0.98
	99.13	117.5	139.75	98.22	119.02	139.821	0.99	1.01	1.00
	82.55	102.25	126.94	85.42	106.22	127.02	1.03	1.04	1.00
Mean							1.01	1.02	1.01
σ							0.09	0.05	0.03
v							0.77	0.22	0.11

#### Equation verification

The proposed equation was verified utilizing the experimental work of Khalil etal^10^. and showed a good agreement, as presented in Table 7.

**Table 7:** Verification of the proposed numerical equation on others’ experimental work.

Specimen		$\:{T}_{u}^{Exp.}$, KN.m	$\:{T}_{u}^{Eqn.}$, KN.m	$\:\frac{{T}_{u}^{Eqn.}}{{T}_{u}^{Exp.}}$
Khalil et al^10^.	GII-C	55.5	57.8	1.04

#### Validity and limitations of the equation

The verification confirmed the equation’s accuracy in the case of small openings and normal-strength concrete. Accordingly, the equation is applicable only to hollow reinforced concrete beams with conventional longitudinal reinforcement, using either normal-or high-strength concrete, and containing a single rectangular opening—whether small or large—at the mid-span on both sides. It is valid when using conventional steel stirrups or GFRP stirrups, provided the following mathematical condition is satisfied: $\:\:305\left(Z\right)$< $\:30\:\left(X\right)+{10}^{-10}\left(Y\right).$.

### Derived equations for solid RC beams

The coefficients of the variables in the first proposed equation for hollow RC beams with opening were modified to develop two additional equations suitable for solid RC. These were adjusted to fit the experimental results of Askar^33^:

9 $$\:{T}_{u}={10}^{6}\left[10\left(X^{\prime}\right)+{2x10}^{-9}(Y^{\prime})-198\left(Z\right)\right],\text{f}\text{o}\text{r}\:Z\hspace{0.17em}<\hspace{0.17em}0.09\:$$

10 $$\:X^{\prime}\:=\:\left(1+0.75\:{\rho\:}_{st}\:\right)$$

11 $$\:Y^{\prime}\:={t}^{3}\left({75\rho\:}_{sl}.{f}_{y}+6000{f}_{c}-{E}_{st}\right)$$

12 $$\:Z\:=\left(\frac{d}{D}x\frac{l}{L}\right)$$

13 $$\:{T}_{u}={10}^{6}\left[6\left(X^{\prime \prime} \right)+{2.5x10}^{-9}\left(Y^{\prime \prime} \right)-11\left(Z\right)\right],\text{f}\text{o}\text{r}\:Z\hspace{0.17em}\ge\:\hspace{0.17em}0.09\:$$

14 $$\:X^{\prime \prime} =\left(1+0.167\:{\rho\:}_{st}\:\right)$$

15 $$\:Y^{\prime \prime} ={t}^{3}\left({12\rho\:}_{sl}.{f}_{y}+7720{f}_{c}-{E}_{st}\right)$$

16 $$\:Z\:=\:\left(\frac{d}{D}x\frac{l}{L}\right)$$

All lengths in (mm) and, all stresses in (MPa), and $\:{\varvec{T}}_{\varvec{u}}$ in (N·mm).

#### Equations verification

A comparison between the outputs of the proposed equations and the experimental results conducted by Askar^33^, Abdel-Kareem and Abd El Salam^4^, Meleka etal^5^., and Abdo and Mabrouk (34) showed reasonable agreement, as summarized in Table 8.

#### Validity and limitations of the equations

This equations are valid only for solid RC beams with conventional stirrups, normal-strength concrete, and conventional longitudinal reinforcement, containing a single rectangular opening located at the mid-span on both sides. The following mathematical conditions must be satisfied: $\:\:198\left(Z\right)$<$\:10\:\left(X^{\prime}\right)+{2x10}^{-9}\left(Y^{\prime}\right)$, $\:11\left(Z\right)<6\left(X^{\prime \prime} \right)+{2.5x10}^{-9}\left(Y^{\prime \prime}\right).$

**Table 8:** Comparison between proposed equations and experimental results for solid beams with web opening.

Specimen		$\:{T}_{u}^{Exp.}$, KN.m	$\:{T}_{u}^{Eqn.}$, KN.m	$\:\frac{{T}_{u}^{Eqn..}}{{T}_{u}^{Exp.}}$
Askar^33^,	D2	33.68	31.47	0.93
	D3	26.40	28.99	1.10
	D4	25.80	26.52	1.03
	D5	22.00	24.04	1.09
	D6	21.00	20.58	0.98
	E2	27.40	25.43	0.93
	E3	21.00	18.01	0.86
	E4	17.12	16.54	0.97
	E5	16.15	16.13	1.00
	E6	13.80	14.44	1.05
	F2	14.40	13.16	0.91
	F3	10.90	10.38	0.95
	F4	8.60	9.69	1.13
	F5	9.20	9.01	0.98
	F6	7.00	6.26	0.89
Mean				0.99
Abdel-Kareem and Abd El Salam^4^	R-2–5 V	6.81	6.40	0.94
	R-2–10 V	6.75	6.92	1.03
	R-4–5 V	6.50	6.20	0.95
	R-4–10 V	7.79	7.95	1.02
	R-2–5 V	6.75	7.43	1.10
	R-2–5 V	7.00	7.44	1.06
	R-4–5 V	7.05	7.36	1.04
Mean				0.97
Meleka et al^5^.	R30*6	5.00	5.15	1.03
	R30*12	4.30	4.69	1.09
	R60*6	4.50	3.66	0.81
	R60*12	3.20	3.64	1.14
Mean				1.02
Abdo and Mabrouk^34^	N1-S125-H300	11.20	12.42	1.11
	N1-S100-H300	12.90	13.19	1.02
	N1-S167-H300	9.76	11.64	1.19
	N1-S125-H350	11.96	12.23	1.02
	N1-S125-H400	14.30	12.51	0.87
Mean				1.04

## Conclusions

This study aimed to quantify the torsional reduction caused by large rectangular web opening in high-strength hollow concrete beams, evaluate the effectiveness of transverse and longitudinal reinforcement in restoring torsional strength, and develop a predictive equation for torsional capacity. Based on the numerical results, the following conclusions are drawn:

1. Effect of opening:
  Large openings decrease torsional capacity by 48.35%, 46.20%, and 37.58% for concrete strengths of 60, 80, and 100 MPa, respectively.
  Opening depth has a greater influence on reducing capacity than its length.
  When (l/L > 0.525) and (d/D > 0.2), additional increases origin a significant drop in torsional resistance.
  *Insight and Implication*: High-strength concrete better redistributes torsional stresses, reducing the impact of openings. To maintain performance, opening depth and ratios should be limited, and additional reinforcement should be provided when these thresholds are exceeded.
2. Transverse reinforcement:
  Increasing stirrup ratio improves torsional capacity, but the benefit decreases with higher concrete strength or longer openings.
  Reducing stirrup spacing below 100 mm is ineffectual, and closer spacing is more valuable than increasing stirrup diameter.
  GFRP stirrups doubled the torsional capacity compared to mild steel or no stirrups, attaining the highest cracking-to-ultimate torque ratio.
  *Insight and Implication*: Optimize spacing rather than diameter; use GFRP near large openings for superior strength and corrosion resistance.
3. Longitudinal reinforcement:
  Increasing longitudinal reinforcement from 1.96% to 2.83% produces higher increases in low-strength beams and those with large openings by of 9.5%, 7.68%, and 5.14% for 60, 80, and 100 MPa, respectively.
  *Insight and Implication*: Most effective in low-strength beams or beams with large openings; critical for compensating torsion loss.
4. Proposed formula:
  The developed equations align closely with existing experimental data, **Insight and Implication**: These formulas can be confidently used for preliminary design to estimate torsional capacity without requiring complex numerical modeling.

### Future work

Further research should explore different opening shapes, multiple openings, combined loading conditions, and alternative reinforcement materials. Besides, for larger openings, use extra reinforcement or alternative structural solutions to offset torsional capacity loss.

## Data Availability

All data generated or analyses during this study are included in this published article.

## References

[1] Meleka, N. N., Mousa, M. A. & Sigutri, M. M. Nonlinear analysis of RC beams with openings subjected to torsion. *ERJ Eng. Res. J.* **30**(3), 375–384 (2007).

[2] Al-Sherrawi, M. H. & Shanshal, Z. M. Torsional resistance of reinforced concrete girders with web openings. *J. Eng.* **22**(2), 137–155 (2016).

[3] Jabbar, S., Hejazi, F. & Mahmod, H. M. Effect of an opening on reinforced concrete Hollow beam web under torsional, flexural, and cyclic loadings. *Latin Am. J. Solids Struct.* **13**(8), 1576–1595 (2016).

[4] Abdel-kareem, A. H. & Salam, A. E. Experimental and analytical investigation of reinforced concrete beams with large web opening under pure torsion. *Int. J. Adv. Eng. Manage. Sci.* **6**(1), 18–33 (2020).

[5] Hekal, G. M., Ramadan, B. A. & Meleka, N. N. Behavior of RC beams with large openings subjected to pure torsion and retrofitted by steel or CFRP plates. *ERJ Eng. Res. J.* **43**(2), 127–138 (2020).

[6] Lisantono, A., Besari, M. S., Suhud, R. & Soemardi, B. W. Experimental investigation on the effect of web opening dimension on the behavior of R/C hybrid deep T-beam subjected to pure torsion. *Jurnal Teknik Sipil ITB*. **11**(1), 1–8 (2004).

[7] Makhlouf, M. Torsional behavior of RC beams with opening using (CFRP-GFRP-steel) stirrups. *Adv. Res.* **8**(3), 1–10 (2016).

[8] Salama, A. E., Kassem, M. E. & Mahmoud, A. A. Torsional behavior of T-shaped reinforced concrete beams with large web openings. *J. Building Eng.* **18**, 84–94 (2018).

[9] Aziz, A. H. & Moatt, A. H. Located torsional box beams with transverse openings. *J. Eng. Sustain. Dev.* (2020).

[10] El-HakimKhalil, A., Etman, E., Atta, A. & Fayed, S. Torsional strengthening of RC box beams using external prestressing technique. *IOSR J. Mech. Civil Eng.* **12**(2), 30–41 (2015).

[11] Alamli, A. S. A. & Abdulameer, S. H. Behavior of hollow I-beams reactive powder concrete with opening for pure torque. *Int. J. Civil Struct. Environ. Infrastruct. Eng. Res. Dev.* ISSN (P): 2249–6866; ISSN (E): 2249–7978 **7**(5), 67–76 (2017).

[12] Mahdi, H. M. & Abbas, R. M. Effect of openings on the torsional behavior of SCC box beams under monotonic and repeated loading. *Civil Eng. J*. **9**(09) (2023).

[13] Eltaly, B., EL_Sayed, M., Meleka, N. & Kandil, K. Torsion behavior of strengthened reinforced concrete box girders with openings: analytical and experimental investigation. *Structures*. **60**, 105908 (2024).

[14] Ibrahim, A., Askar, H. S. & El-Zoughiby, M. E. Experimental and numerical nonlinear analysis of hollow RC beams reinforced with rectangular spiral stirrups under torsion. *Iran. J. Sci. Technol. Trans. Civil Eng.* **46**(6), 4019–4029 (2022).

[15] Mahdi, H. M. & Abbas, R. M. Torsional behavior of CFRP strengthening of SCC box beams with web openings under repeated loading. *Civil Eng. J.* **9**(12), 3038–3059 (2023).

[16] El-Basiouny, A. M., Askar, H. S. & El-Zoughiby, M. E. Effect of compression pre-force and web openings on torsional strength of UHPC hollow beams using numerical and mathematical modeling. *Sci. Rep.* **15**(1), 25880 (2025). 40670528 10.1038/s41598-025-10834-0 PMC12267853

[17] Mansur, M. A., Ting, S. K. & Lee, S. L. Torsion tests of r/c beams with large openings. *J. Struct. Eng.* **109**(8), 1780–1791 (1983).

[18] Ahmed, M. *Torsional behavior of concrete beams of solid and hollow sections reinforced with inclined spirals*. PhD thesis, Mansoura University, Egypt Archived in the Egyptian Universities Libraries Consortium. (2021).

[19] Lopes, S. M. R. & Bernardo, L. F. A. Twist behavior of high-strength concrete hollow beams–formation of plastic hinges along the length. *Eng. Struct.* **31**(1), 138–149 (2009).

[20] William, K. J. & Warnke, E. P. Constitutive model for the triaxial behavior of concrete. In *Proceedings, International Association for Bridge and Structural Engineering*. Vol. 19, 174 (ISMES, 1975).

[21] ANSYS, Verification Manual, Release 15.0. (ANSYS Inc., 2013).

[22] ACI 318–19, Building Code Requirements for Reinforced Concrete and Commentary. (2019).

[23] Desayi, P. & Krishnan, S. Equation for the stress-strain curve of concrete. *ACI J.*. Proc. 61. 10.14359/7785 (1964).

[24] Cervenka, V., Eligehausen, R. & Pukl, R. SBETA: computer program for nonlinear finite element analysis of reinforced concrete structures;Bericht Nr. 4/9–89/22, Institut für Werkstoffe Im Bauwesen. IWB. (1990).

[25] Ahmad, H. et al. Finite element analysis of glass fiber-reinforced polymer-(Gfrp) reinforced continuous concrete beams. *Polymers* **13**(24), 4468 (2021). 34961019 10.3390/polym13244468 PMC8708554

[26] Al Omar, S. & Abdelhadi, A. Comparative life-cycle assessment of steel and GFRP rebars for procurement sustainability in the construction industry. *Sustainability* **16**(10), 3899 (2024).

[27] Ascione, F., Maselli, G. & Nesticò, A. Sustainable materials selection in industrial construction: A life-cycle based approach to compare the economic and structural performances of glass fibre reinforced polymer (GFRP) and steel. *J. Clean. Prod.* **475**, 143641 (2024).

[28] Younis, A., Ebead, U. & Judd, S. Life cycle cost analysis of structural concrete using seawater, recycled concrete aggregate, and GFRP reinforcement. *Constr. Build. Mater.* **175**, 152–160 (2018).

[29] Jin, Q. et al. Tensile strength and degradation of Gfrp bars under combined effects of mechanical load and alkaline solution. *Materials* **13**(16), 3533 (2020). 32796501 10.3390/ma13163533 PMC7476013

[30] Tu, J., Xie, H. & Gao, K. Prediction of the long-term performance and durability of GFRP bars under the combined effect of a sustained load and severe environments. *Materials* **13**(10), 2341 (2020). 32438774 10.3390/ma13102341 PMC7287940

[31] Feng, S. Z. et al. Accelerated aging tests of large-diameter GFRP bars in alkaline environment. *Compos. Part. C: Open. Access.* **14**, 100486 (2024).

[32] Myers, J. J. & Al-Khafaji, A. Durability of GFRP bar reinforcement extracted from In-service concrete structures. Research on Concrete Applications for Sustainable Transportation (RE-CAST), (UTC). (No. 00059416) (2020).

[33] Askar, S. H. Behavior of RC beams with web opening subjected to pure torsion. In *Proceedings of International Structural Engineering and Construction*, 10.14455/ISEC.2022.9(1).STR-53 (2022).

[34] Abdo, T. & Mabrouk, R. Effect of web openings on the structural behavior of RC beams subjected to pure torsion. In *MATEC Web of Conferences*, Vol. 120, 01007 (EDP Sciences, 2017).

